# Rain, rain, go away, come again another day: do climate variations enhance the spread of COVID-19?

**DOI:** 10.1186/s12992-024-01044-w

**Published:** 2024-05-14

**Authors:** Masha Menhat, Effi Helmy Ariffin, Wan Shiao Dong, Junainah Zakaria, Aminah Ismailluddin, Hayrol Azril Mohamed Shafril, Mahazan Muhammad, Ahmad Rosli Othman, Thavamaran Kanesan, Suzana Pil Ramli, Mohd Fadzil Akhir, Amila Sandaruwan Ratnayake

**Affiliations:** 1 Faculty of Maritime Studies, Universiti Malaysia Terengganu, 21030 Kuala Nerus, Terengganu Malaysia; 2 Institute of Oceanography and Environment, Universiti Malaysia Terengganu, 21030 Kuala Nerus, Terengganu Malaysia; 3 Faculty of Science and Marine Environment, Universiti Malaysia Terengganu, 21030 Kuala Nerus, Terengganu Malaysia; 4 Institute for Social Science Studies, Universiti Putra Malaysia, 43400 Serdang, Selangor Malaysia; 5 Social, Environmental and Developmental Sustainability Research Center, Faculty of Social Sciences and Humanities, Universiti Kebangsaan Malaysia, 43600 Bangi, Selangor Malaysia; 6 Institute of Geology Malaysia, Board of Geologists, 62100 Putrajaya, Malaysia; 7 Executive Office, Proofreading By A UK PhD, 51-1, Biz Avenue II, 63000 Cyberjaya, Malaysia; 8 Faculty of Engineering, Universiti Malaya, 50603 Kuala Lumpur, Malaysia; 9 Faculty of Applied Sciences, Uva Wellassa University, Badulla, 90000 Sri Lanka

**Keywords:** Coronavirus, Solar radiation, Temperature, Humidity, Social distancing

## Abstract

**Abstract:**

The spread of infectious diseases was further promoted due to busy cities, increased travel, and climate change, which led to outbreaks, epidemics, and even pandemics. The world experienced the severity of the 125 nm virus called the coronavirus disease 2019 (COVID-19), a pandemic declared by the World Health Organization (WHO) in 2019. Many investigations revealed a strong correlation between humidity and temperature relative to the kinetics of the virus’s spread into the hosts. This study aimed to solve the riddle of the correlation between environmental factors and COVID-19 by applying RepOrting standards for Systematic Evidence Syntheses (ROSES) with the designed research question. Five temperature and humidity-related themes were deduced via the review processes, namely 1) The link between solar activity and pandemic outbreaks, 2) Regional area, 3) Climate and weather, 4) Relationship between temperature and humidity, and 5) the Governmental disinfection actions and guidelines. A significant relationship between solar activities and pandemic outbreaks was reported throughout the review of past studies. The grand solar minima (1450-1830) and solar minima (1975-2020) coincided with the global pandemic. Meanwhile, the cooler, lower humidity, and low wind movement environment reported higher severity of cases. Moreover, COVID-19 confirmed cases and death cases were higher in countries located within the Northern Hemisphere. The Blackbox of COVID-19 was revealed through the work conducted in this paper that the virus thrives in cooler and low-humidity environments, with emphasis on potential treatments and government measures relative to temperature and humidity.

**Highlights:**

• The coronavirus disease 2019 (COIVD-19) is spreading faster in low temperatures and humid area.

• Weather and climate serve as environmental drivers in propagating COVID-19.

• Solar radiation influences the spreading of COVID-19.

• The correlation between weather and population as the factor in spreading of COVID-19.

**Graphical abstract:**

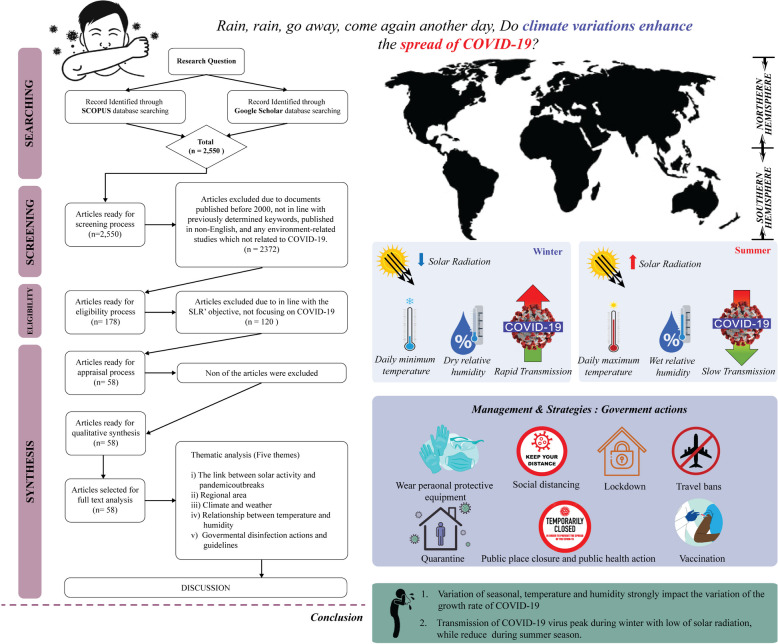

## Introduction

**Fig. 1:**
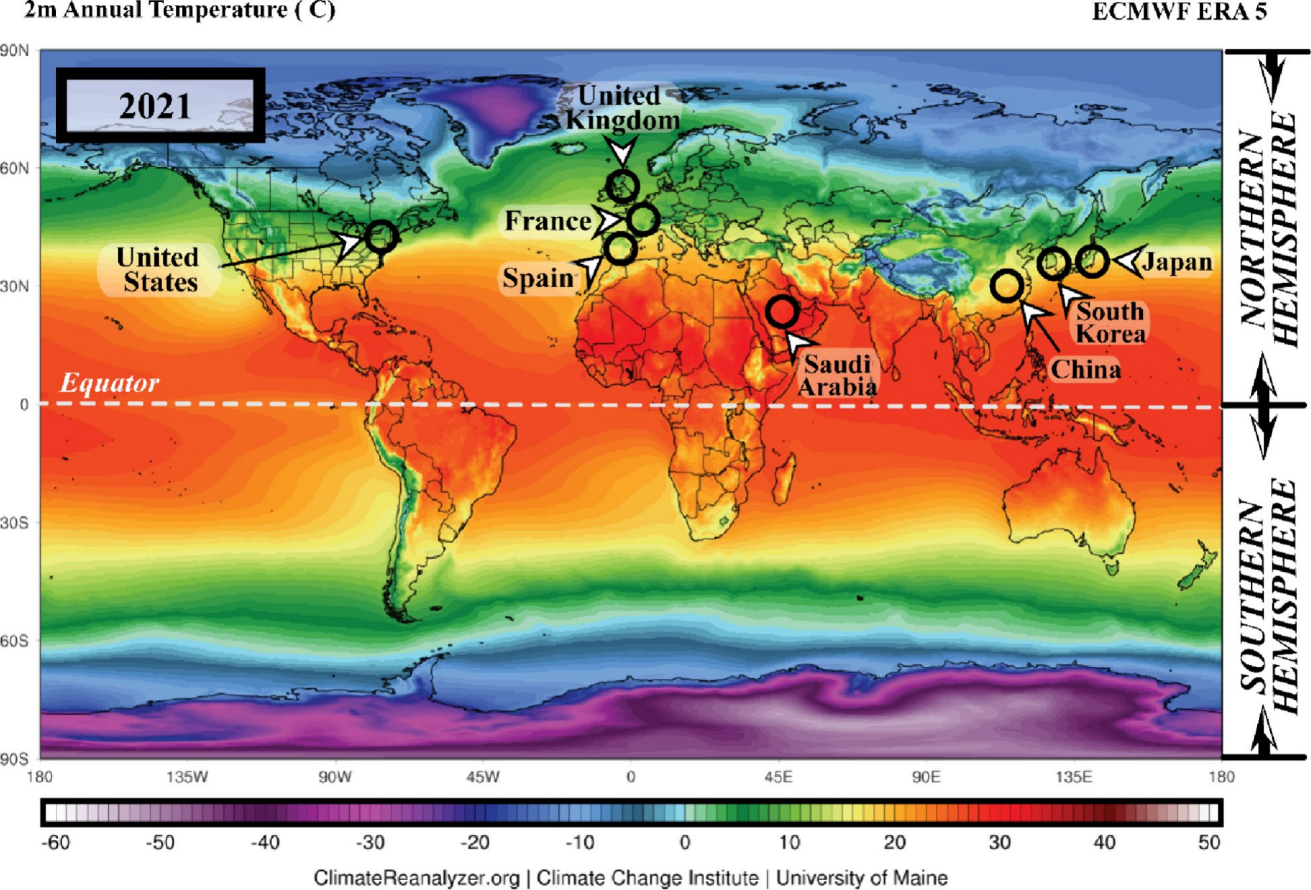
The annual average temperature data for 2021 in the northern and southern hemispheres (*Source:* meteoblue.com). *Note:* The black circles mark countries with high Coronavirus disease 2019 (COVID-19) infections

The revolution and rotation of the Earth and the Sun supply heat and create differential heating on earth. The movements and the 23.5° inclination of the Earth [1] separate the oblate-ellipsoid-shaped earth into northern and southern hemispheres. Consequently, the division results in various climatic zones at different latitudes and dissimilar local temperatures (see Fig. 1) and affects the seasons and length of a day and night in a particular region [2]. Global differential heating and climate variability occur due to varying solar radiation received by each region [3]. According to Trenberth and Fasullo [4] and Hauschild et al. [5] the new perspective on the issue of climate change can be affected relative to the changes in solar radiation patterns. Since the study by Trenberth and Fasullo [4] focused on climate model changes from 1950 to 2100, it was found that the role of changing clouds and trapped sunlight can lead to an opening of the aperture for solar radiation.

**Fig. 2:**
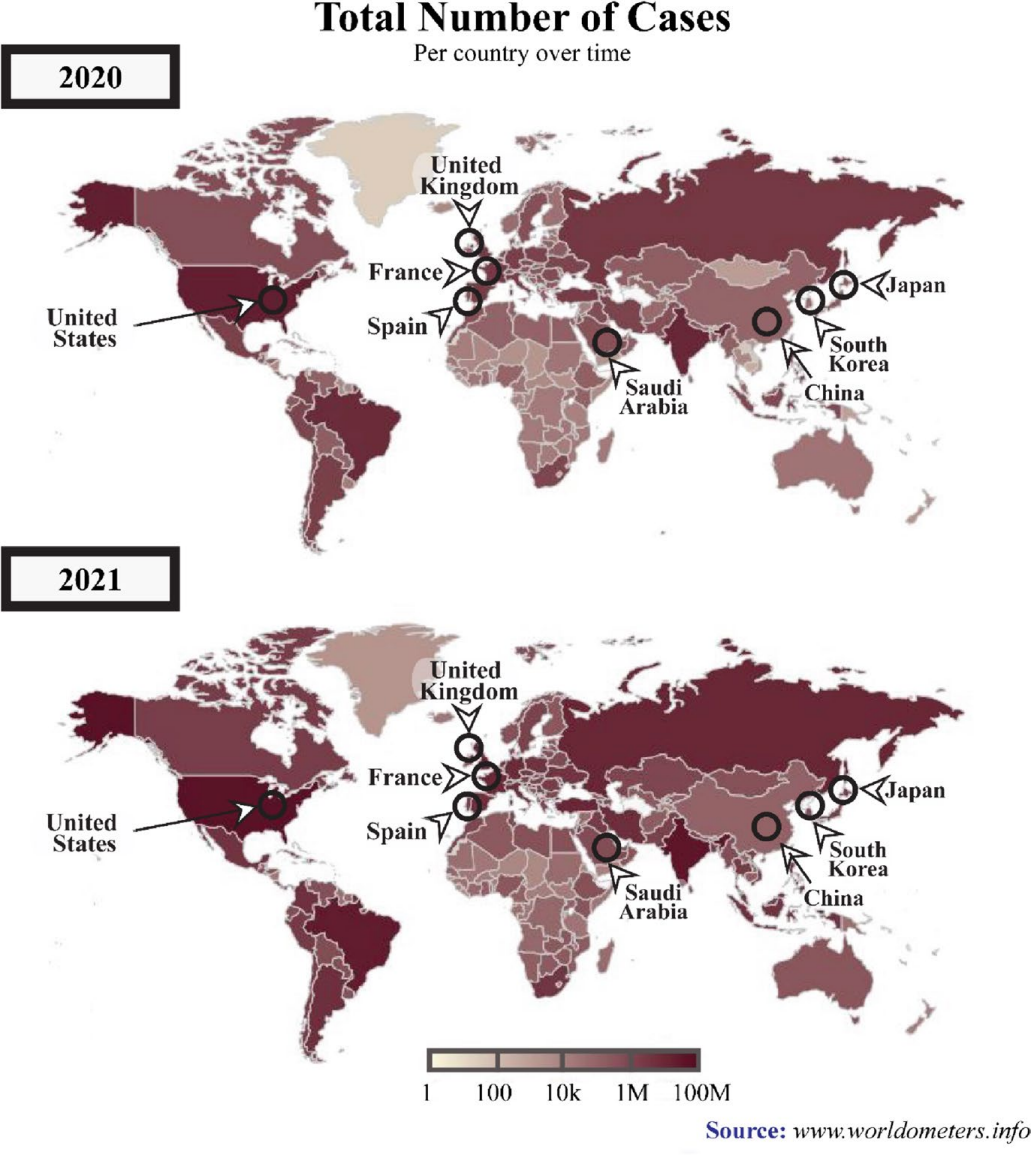
A graphical representation of the total number of COVID-19 cases across various periods between 2020 and 2021. (*Source*: www.worldometers.info). *Note:* The black circles indicate countries with high numbers COVID-19-infections

Furthermore, the heat from sunlight is essential to humans; several organisms could not survive without it. Conversely, the spread of any disease-carrying virus tends to increase with less sunlight exposure [6]. Historically, disease outbreaks that led to epidemic and pandemic eruptions were correlated to atmospheric changes. Pandemic diseases, such as the flu (1918), Asian flu (1956–1958), Hong Kong flu (1968), and recently, the coronavirus disease 2019 (COVID-19) (2019), recorded over a million death toll each during the winter season or minimum temperature conditions [7]. The total number of COVID-19 cases is illustrated in Fig. 2.

In several previous outbreaks, investigations revealed a significant association between temperature and humidity with a particular focus on the transmission dynamics of the infection from the virus into the hosts [8–10]. Moreover, disease outbreaks tended to heighten in cold temperatures and low humidity [11]. Optimal temperature and sufficient relative humidity during evaporation are necessary for cloud formation, resulting in the precipitated liquid falling to the ground as rain, snow, or hail due to the activity of solar radiation balancing [4].

Consequently, the radiation balancing processes in the atmosphere are directly linked to the living beings on the earth, including plants and animals, and as well as viruses and bacterias. According to Carvalho et al. [12]‘s study, the survival rate of the Coronaviridae Family can decrease during summer seasons. Nevertheless, numerous diseases were also developed from specific viruses, such as influenza, malaria, and rubella, and in November 2019, a severe health threat originated from a 125 nm size of coronavirus, had resulted in numerous deaths worldwide.

### Transmission and symptoms of COVID-19

The COVID-19, or severe acute respiratory syndrome coronavirus 2 (SARS-CoV-2), is an infectious disease caused by a newly discovered pathogenic virus from the coronavirus family, the novel coronavirus (2019-nCoV) [13]. The first case was recorded in Wuhan, China, in December 2019 [14]. The pathogenic virus is transmitted among humans when they breathe in air contaminated with droplets and tiny airborne particles containing the virus [14–18].

According to the World Health Organization (WHO), the most common symptoms of COVID-19 infection include fever, dry cough, and tiredness. Nevertheless, older people and individuals with underlying health problems (lung and heart problems, high blood pressure, diabetes, or cancer) are at higher risk of becoming seriously ill and developing difficulty breathing [19]. The COVID-19 was initially only predominant in China but rapidly spread to other countries globally. The remarkably swift acceleration of the number of infections and mortality forced WHO to declare COVID-19 a global public health emergency on the 30th of January 2020, which was later declared as a pandemic on the 11th of March 2020 [20].

Since no vaccine was available then, WHO introduced the COVID-19 preventative measures to reduce the chances of virus transmission. The guideline for individual preventative included practising hand and respiratory hygiene by regularly cleaning hands with soap and water or alcohol-based sanitisers, wear a facemask and always maintaining at least a one-meter physical distance [21]. Nevertheless, the worldwide transmission of COVID-19 has resulted in fear and forced numerous countries to impose restrictions rules, such as lockdown, travel bans, closed country borders, restrictions on shipping activities, and movement limitations, to diminish the spread of COVID-19 [22].

According to WHO, by the 2nd of December 2020, 63,379,338 confirmed cases and 1,476,676 mortalities were recorded globally. On the 3rd of December 2021, 263,655,612 confirmed cases and deaths were recorded, reflecting increased COVID-19 infections compared to the previous year. The American and European regions documented the highest COVID-19 patients with 97,341,769 and 88,248,591 cases, respectively (see Fig. 2), followed by Southeast Asia with 44,607,287, Eastern Mediterranean accounted 16,822,791, Western Pacific recorded 6,322,034, and Africa reported the lowest number of cases at 6,322,034 [19].

Recently, an increasing number of studies are investigating the association between environmental factors (temperature and humidity) and the viability, transmission, and survival of the coronavirus [23–26]. The results primarily demonstrated that temperature was more significantly associated with the transmission of COVID-19 [27–29] and its survival period on the surfaces of objects [30]. Consequently, the disease was predominant in countries with low temperature and humidity [31], which was also proven by Diao et al. [32]‘s study demonstrating higher rates of COVID-19 transmission in China, England, Germany, and Japan.

A comprehensive systematic literature review (SLR) is still lacking despite numerous research on environmental factors linked to coronavirus. Accordingly, this article aimed to fill the gap in understanding and identifying the correlation between environmental factors and COVID-19 by analysing existing reports. Systematically reviewing existing literature is essential to contribute to the body of knowledge and provide beneficial information for public health policymakers.

## Methodology

The present study reviewed the protocols, formulation of research questions, selection of studies, appraisal of quality, and data abstraction and analysis.

### The protocol review

**Fig. 3:**
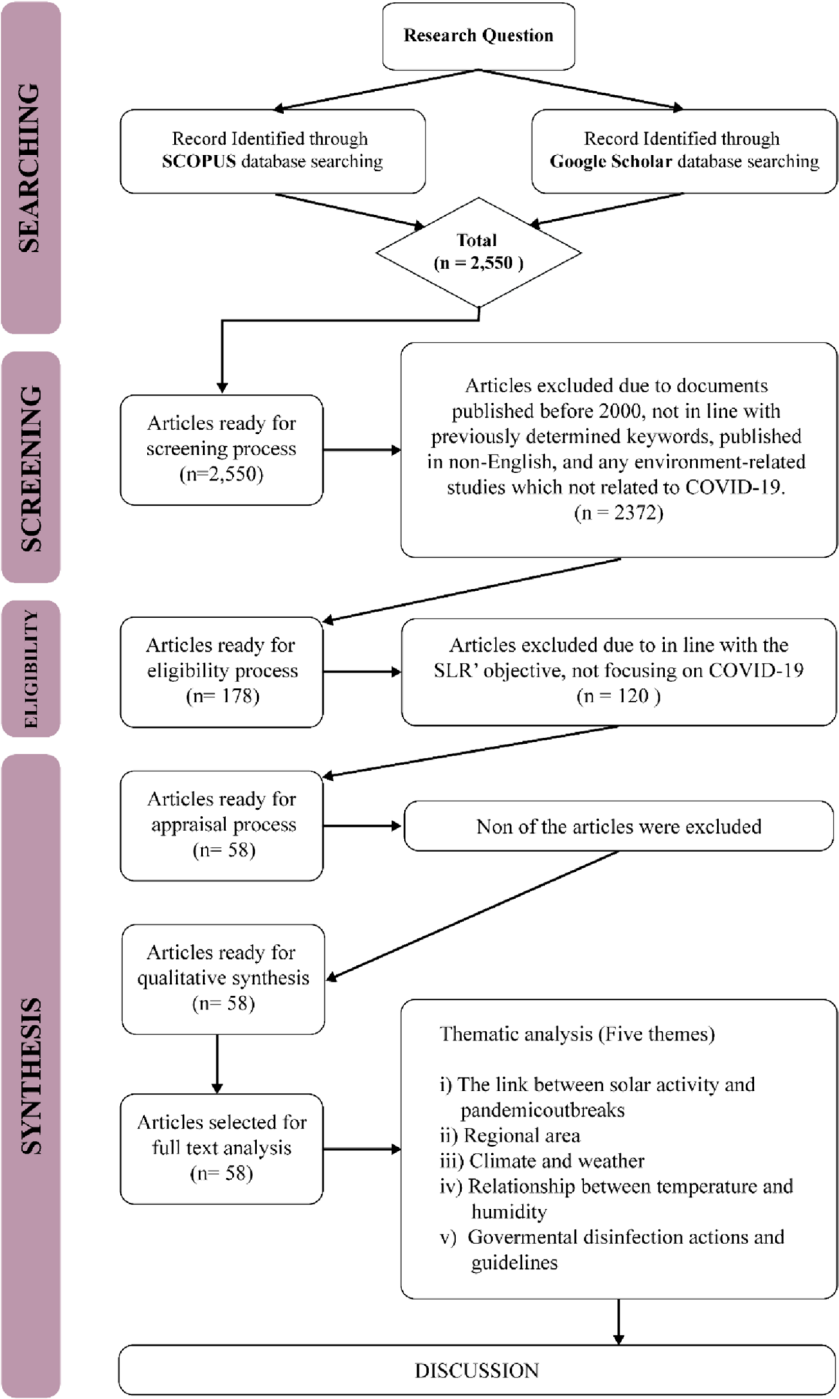
The flow diagram guide by ROSES protocol and Thematical Analysis

The present SLR was performed according to the reporting standards for systematic evidence syntheses (ROSES) and followed or adapted the guidelines as closely as possible. Thus, in this study, a systematic literature review was guided by the ROSES review protocol (Fig. 3). Compared to preferred reporting items for systematic review and meta-analysis (PRISMA), ROSES is a review protocol specifically designed for a systematic review in the conservation or environment management fields [33]. Compared to PRISMA, ROSES offers several advantages, as it is tailored to environmental systematic review, which reduces emphasis on quantitative synthesis (e.g. meta-analysis etc.) that is only reliable when used with appropriate data [34].

The current SLR started by determining the appropriate research questions, followed by the selection criteria, including the review, specifically on the keywords employed and the selection of journals database. Subsequently, the appraisal quality process and data abstraction and analysis were conducted.

### Formulation of research questions

The entire process of this SLR was guided by the specific research questions, while sources to be reviewed and data abstraction and analysis were in line with the determined research question [35, 36]. In the present article, a total of five research questions were formed, namely:

1. What the link between solar activity and COVID-19 pandemic outbreaks?
2. Which regions were more prone to COVID-19?
3. What were the temporal and spatial variabilities of high temperature and humidity during the spread of COVID-19?
4. What is the relationship between temperature and humidity in propagating COVID-19?
5. How did the government’s disinfection actions and guidelines can be reducing the spread of COVID-19?

### Systematic searching strategies

#### Selection of studies

In this stage of the study, the appropriate keywords to be employed in the searching process were determined. After referring to existing literature, six main keywords were chosen for the searching process, namely COVID-19, coronavirus, temperature, humidity, solar radiation and population density. The current study also utilised the boolean operators (OR, AND, AND NOT) and phrase searching.

Scopus was employed as the main database during the searching process, in line with the suggestion by Gusenbauer and Haddaway [37], who noted the strength of the database in terms of quality control and search and filtering functions. Furthermore, Google Scholar was selected as the supporting database. Although Halevi et al. [38] expressed concerns about its quality, Haddaway et al. [39] reported that due to its quantity, Google Scholar was suitable as a supporting database in SLR studies.

In the first stage of the search, 2550 articles were retrieved, which were then screened. The suitable criteria were also determined to control the quality of the articles reviewed [40]. The criteria are: any documents published between 2000 to 2022, documents that consist previously determined keywords, published in English, and any environment-related studies that focused on COVID-19. Based on these criteria, 2372 articles were excluded and 178 articles were proceeded to the next step namely eligibility. In the eligibility process, the title and the abstract of the articles were examined to ensure its relevancy to the SLR and in this process a total of 120 articles were excluded and only 58 articles were processed in the next stage.

#### Appraisal of the quality

The study ensured the rigor of the chosen articles based on best evidence synthesis. In the process, predefined inclusion criteria for the review were appraised by the systematic review team based on previously established guidelines and the studies were then judged as being scientifically admissible or not [40]. Hence, by controlling the quality based on the best evidence synthesis, the present SLR controls its quality by including articles that are in line with the inclusion criteria. It means that any article published within the timeline (in the year 2000 and above), composed of predetermined keywords, in English medium, and environment-related investigations focusing on COVID-19 are included in the review. Based on this process, all 58 articles fulfilled all the inclusion criteria and are considered of good quality and included in the review.

#### Data abstraction and analysis

The data abstraction process in this study was performed based on five research questions (please refer to 2.2, formulation of research questions). The data that was able to answer the questions were abstracted and placed in a table to ease the data analysis process. The primary data analysis technique employed in the current study was qualitative and relied on thematic analysis.

The thematic technique is a descriptive method that combines data flexibly with other information evaluation methods [41], aiming to identify the patterns in studies. Any similarities and relationships within the abstracted data emerge as patterns. Subsequently, suitable themes and sub-themes would be developed based on obtained patterns [42]. Following the thematic process, five themes were selected in this study.

## Results

### Background of the selected articles

The current study selected 58 articles for the SLR. Five themes were developed based on the thematic analysis from the predetermined research questions: the link between solar activity and pandemic outbreaks, regional area, climate and weather, the relationship between temperature and humidity, and government disinfection action guidelines. Among the articles retrieved between 2000 and 2022; two were published in 2010, one in 2011, four in 2013, three in 2014, two in 2015, six in 2016 and 2017, respectively, one in 2018, six in 2019, twelve in 2020, eight in 2021, and seven in 2022.

### Temperature- and humidity-related themes

#### The link between solar activity and pandemic outbreaks

Numerous scientists have investigated the relationship between solar activities and pandemic outbreaks over the years ([43]; A [27, 44, 45].). Nuclear fusions from solar activities have resulted in minimum and maximum solar sunspots. Maximum solar activities are characterised by a high number of sunspots and elevated solar flare frequency and coronal mass injections. Minimum solar sunspot occurrences are identified by low interplanetary magnetic field values entering the earth [1].

A diminished magnetic field was suggested to be conducive for viruses and bacteria to mutate, hence the onset of pandemics. Nonetheless, Hoyle and Wickramasinghe [46] reported that the link between solar activity and pandemic outbreaks is only speculative. The literature noted that the data recorded between 1930 and 1970 demonstrated that virus transmissions and pandemic occurrences were coincidental. Moreover, no pandemic cases were reported in 1979, when minimum solar activity was recorded [47].

Chandra Wickramasinghe et al. [48] suggested a significant relationship between pandemic outbreaks and solar activities as several grand solar minima, including Sporer (1450–1550 AD), Mounder (1650–1700 AD), and Dalton (1800–1830) minimums, were recorded coinciding with global pandemics of diseases, such as smallpox, the English sweat, plague, and cholera pandemics. Furthermore, since the Dalton minimum, which recorded minimum sunspots, studies from 2002 to 2015 have documented the reappearance of previous pandemics. For example, influenza subtype H1N1 1918/1919 episodically returned in 2009, especially in India, China, and other Asian countries. Zika virus, which first appeared in 1950, flared and became endemic in 2015, transmitted sporadically, specifically in African countries. Similarly, SARS-CoV was first recorded in China in 2002 and emerged as an outbreak, MERS-CoV, in middle east countries a decade later, in 2012.

In 2020, the World Data Centre Sunspot Index and Long-term Solar Observations (http://sidc.be) confirmed that a new solar activity was initiated in December 2019, during which a novel coronavirus pandemic also occurred, and present a same as the previous hypothesis. Nevertheless, a higher number of pandemic outbreaks were documented during low minimum solar activities, including Ebola (1976), H5N1 (Nipah) (1967–1968), H1N1 (2009), and COVID-19 (2019–current). Furthermore, Wickramasinghe and Qu [49] reported that since 1918 or 1919, more devastating and recurrent pandemics tend to occur, particularly after a century. Consequently, within 100 years, a sudden surge of influenza was recorded, and novel influenza was hypothesised to emerge.

**Fig. 4:**
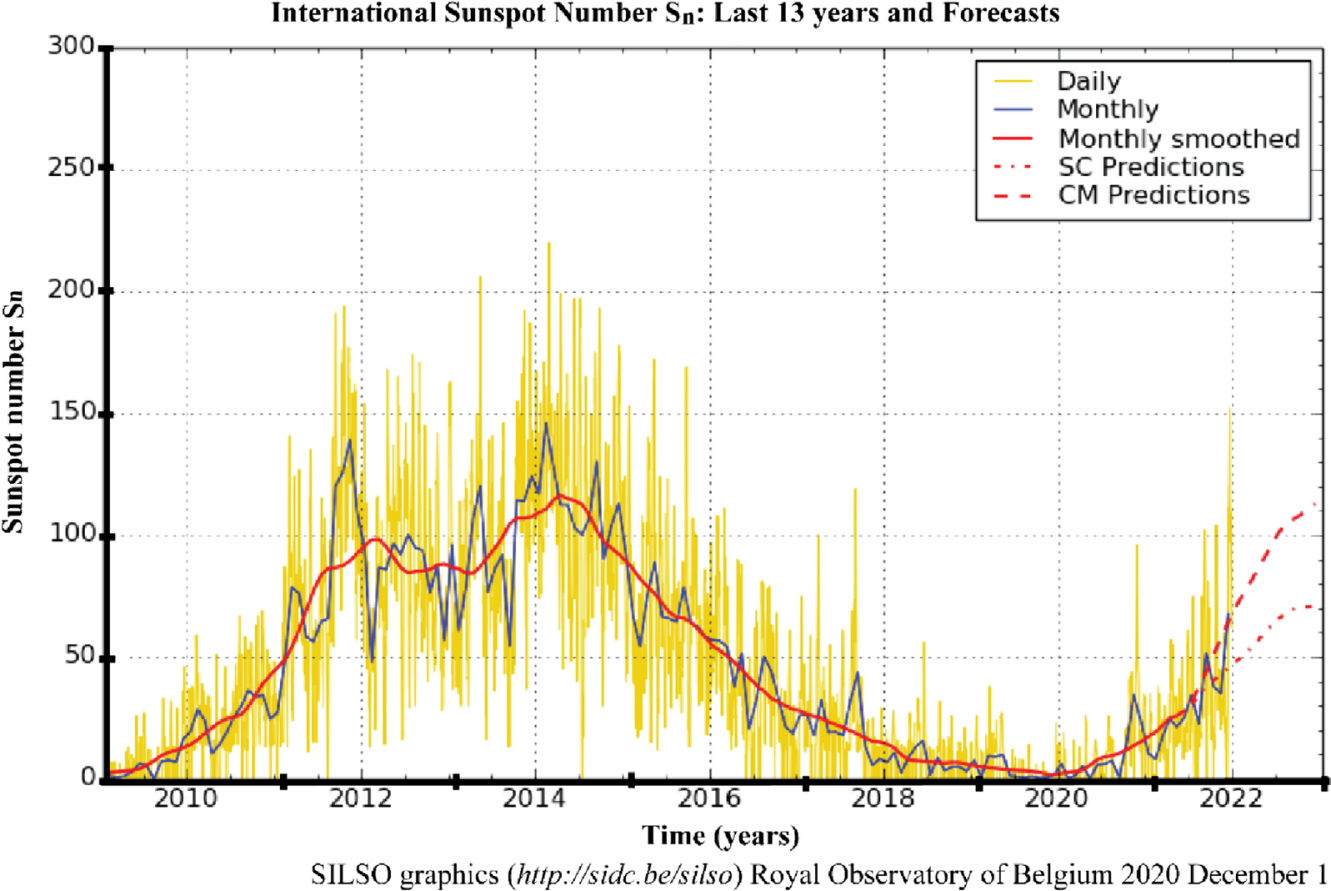
The number of sunspots in the last 13 years. *Note*: The yellow curve indicates the daily sunspot number and the 2010–2021 delineated curve illustrates the minimum solar activity recorded (source: http://sidc.be/silso)

Figure 4 demonstrates that low minimum solar activity significantly reduced before 2020, hence substantiating the claim that pandemic events are closely related to solar activities. Moreover, numerous studies (i.e. [43], Chandra [46–48]) reported that during solar minimums, new viruses could penetrate the surfaces of the earth and high solar radiation would result in lower infection rates, supporting the hypothesis mentioned above.

#### Regional area

In early December 2019, Wuhan, China, was reported as the centre of the severe acute respiratory syndrome coronavirus 2 (SARS-CoV-2) outbreak [50]. Chinese health authorities immediately investigated and controlled the spread of the disease. Nevertheless, by late January 2020, the WHO announced that COVID-19 was a global public health emergency. The upgrade was due to the rapid rise in confirmed cases, which were no longer limited to Wuhan [28]. The disease had spread to 24 other countries, which were mainly in the northern hemisphere, particularly the European and Western Pacific regions, such as France, United Kingdom, Spain, South Korea, Japan, Malaysia, and Indonesia [51, 52]. The migration or movement of humans was the leading agent in the spread of COVID-19, resulting in an almost worldwide COVID-19 pandemic [53].

The first hotspots of the epidemic outspread introduced by the Asian and Western Pacific regions possessed similar winter climates with an average temperature and humidity rate of 5–11 °C and 47–79%. Consequently, several publications reviewed in the current study associated the COVID-19 outbreak with regional climates (i.e. [1, 29, 54, 55]) instead of its close connection to China. This review also discussed the effects of a range of specific climatological variables on the transmission and epidemiology of COVID-19 in regional climatic conditions.

**Fig. 5:**
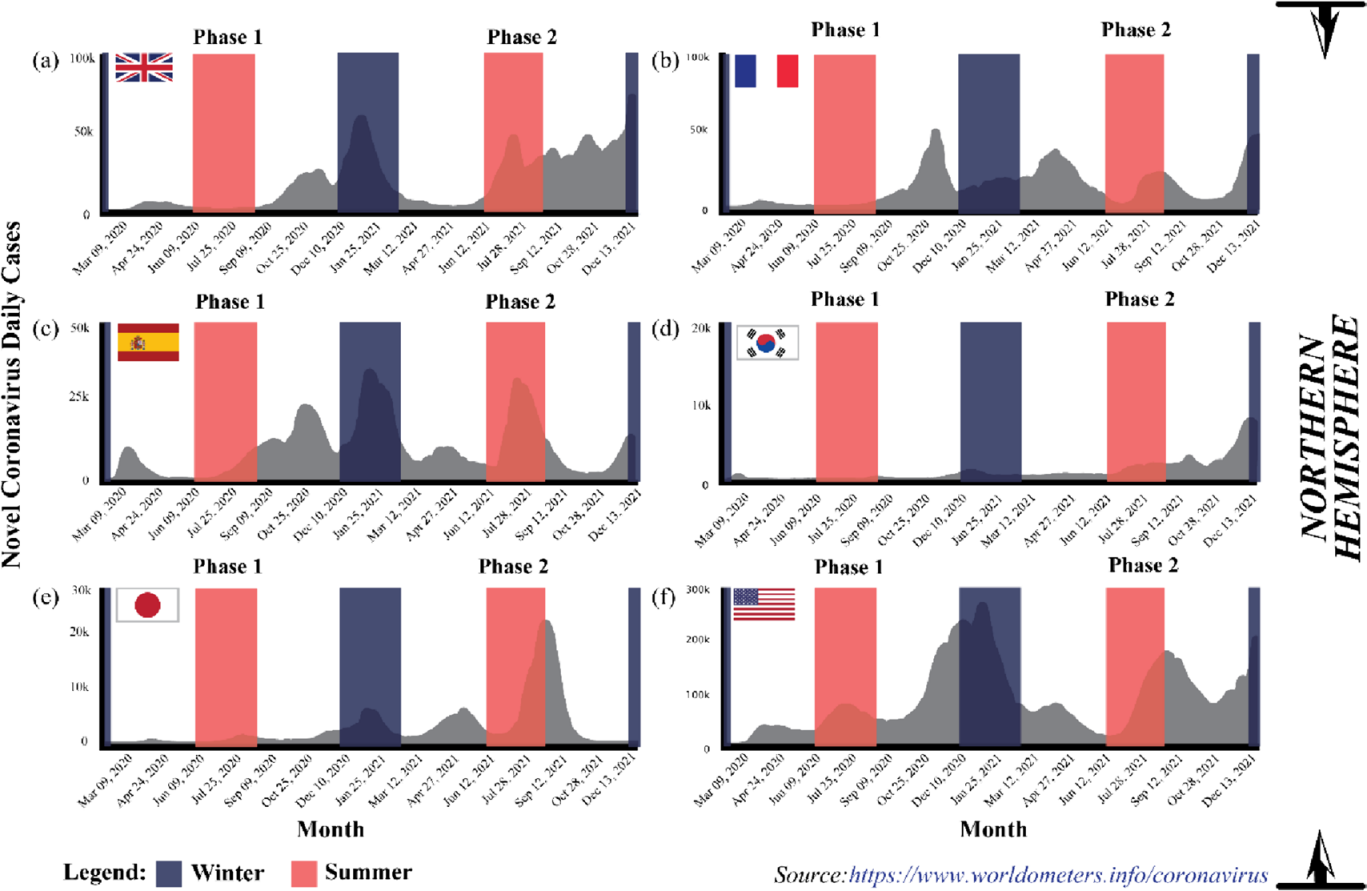
The evolution of the COVID-19 pandemic from the 15th of February 2020 to the 2nd of December 2020 (*Source:* https://www.worldometers.info/coronavirus)

America and Europe documented the highest COVID-19 cases, outnumbering the number reported in Asia [19] and on the 2nd of December 2020, the United States of America (USA) reported the highest number of confirmed COVID-19 infections, with over 13,234,551 cases and 264,808 mortalities (Da S [56].). The cases in the USA began emerging in March 2020 and peaked in late November 2020, during the wintertime in the northern hemisphere (December to March) [53]. Figure 5 demonstrates the evolution of the COVID-19 pandemic in several country which represent comparison two phase of summer and one phase of winter. Most of these countries tend to increase of COVID cases close to winter season. Then, it can be worsening on phase two of summer due to do not under control of human movement although the normal trend it is presenting during winter phase.

The coronavirus spread aggressively across the European region, which recorded the second highest COVID-19 confirmed cases after America. At the end of 2020, WHO reported 19,071,275 Covid-19 cases in the area, where France documented 2,183,275 cases, the European country with the highest number of confirmed cases, followed by the United Kingdom (1,629,661 cases) and Spain (1,652,801 cases) [19]. Europe is also located in the northern hemisphere and possesses a temperate climate.

The spatial and temporal transmission patterns of coronavirus infection in the European region were similar to America and the Eastern Mediterranean, where the winter season increased COVID-19 cases. Typically, winter in Europe occurs at the beginning of October and ends in March. Hardy et al. [57] also stated that temperature commonly drops below freezing (approximately − 1 °C) when snow accumulates between December to mid-March, resulting in an extreme environment. Figure 5 indicates that COVID-19 cases peaked in October when the temperature became colder [21]. Similarly, the cases were the highest in the middle of the year in Australia and South Asian countries, such as India, that experience winter and monsoon, respectively, during the period.

In African regions, the outbreak of COVID-19 escalated rapidly from June to October before falling from October to March, as summer in South Africa generally occurs from November to March, while winter from June to August. Nevertheless, heavy rainfall generally transpires during summer, hence the warm and humid conditions in South Africa and Namibia during summer, while the opposite happens during winter (cold and dry). Consequently, the outbreak in the region recorded an increasing trend during winter and subsided during the summer, supporting the report by Gunthe et al. [58]. Novel coronavirus disease presents unique and grave challenges in Africa, as it has for the rest of the world. However, the infrastructure and resources have limitations for Africa countries facing COVID-19 pandemic and the threat of other diseases [59].

Conclusively, seasonal and regional climate patterns were associated with COVID-19 outbreaks globally. According to Kraemer et al. [60], they used real-time mobility data in Wuhan and early measurement presented a positive correlation between human mobility and spread of COVID-19 cases. However, after the implementation of control measures, this correlation dropped and growth rates became negative in most locations, although shifts in the demographics of reported cases were still indicative of local chains of transmission outside of Wuhan.

#### Climate and weather

The term “weather” represents the changes in the environment that occur daily and in a short period, while “climate” is defined as atmospheric changes happening over a long time (over 3 months) in specific regions. Consequently, different locations would experience varying climates. Numerous reports suggested climate and weather variabilities as the main drivers that sped or slowed the transmission of SARS-CoV-2 worldwide [44, 61–63].

From a meteorological perspective, a favourable environment has led to the continued existence of the COVID-19 virus in the atmosphere [64]. Studies demonstrated that various meteorological conditions, such as the rate of relative humidity (i.e. [28]), precipitation (i.e. [65]), temperature (i.e. [66]), and wind speed factors (i.e. [54]), were the crucial components that contributed to the dynamic response of the pandemic, influencing either the mitigation or exacerbation of novel coronavirus transmission. In other words, the environment was considered the medium for spreading the disease when other health considerations were put aside. Consequently, new opinions, knowledge, and findings are published and shared to increase awareness, thus encouraging preventive measures within the public.

The coronavirus could survive in temperatures under 30 °C with a relative humidity of less than 80% [67], suggesting that high temperatures and lower relative humidity contributed to the elicitation of COVID-19 cases [18, 51, 58, 68]. Lagtayi et al. [7] highlighted temperature as a critical factor, evidently from the increased transmission rate of MERS-Cov in African states with a warm and dry climate. Similarly, the highest COVID-19 cases were recorded in dry temperate regions, especially in western Europe (France and Spain), China, and the USA, while the countries nearer to the equator were less affected. Nevertheless, the temperature factor relative to viral infections depends on the protein available in the viruses. According to Chen and Shakhnovich [69], there is a good correlation between decreasing temperature and the growth of proteins in virus. Consequently, preventive measures that take advantage of conducive environments for specific viruses are challenging.

Precipitation also correlates with influenza [43]. A report demonstrated that regions with at least 150 mm of monthly precipitation threshold level experienced fewer cases than regions with lower precipitation rates. According to Martins et al. [70], influenza and COVID-19 can be affected by climate, where virus can be spread through the respiratory especially during rainfall season. The daily spread of Covid-19 cases in tropical countries, which receive high precipitation levels, are far less than in temperate countries [27]. Likewise, high cases of COVID-19 were reported during the monsoon season (mid-year) in India during which high rainfall is recorded [71]. Moreover, the majority of the population in these regions has lower vitamin D levels, which may contribute to weakened immune responses during certain seasons [27].

Rainfall increases the relative atmospheric humidity, which is unfavourable to the coronaviruses as its transmission requires dry and cold weather. Moreover, several reports hypothesised that rain could wash away viruses on object surfaces, which is still questioned. Most people prefer staying home on rainy days, allowing less transmission or close contact. Conversely, [72] exhibited that precipitation did not significantly impact COVID-19 infectiousness in Oslo, Norway due the location in northern hemisphere which are during winter season presenting so cold.

Coşkun et al. [54] and Wu et al. [29] claimed that wind could strongly correlate with the rate of COVID-19 transmission. Atmospheric instability (turbulent occurrences) leads to increased wind speed and reduces the dispersion of particulate matter (PM_2.5_ and PM_10_) in the environment and among humans. An investigation performed in 55 cities in Italy during the COVID-19 outbreak proved that the areas with low wind movement (stable atmospheric conditions) possessed a higher correlation coefficient and exceeded the threshold value of the safe level of PM_2.5_ and PM_10_. Resultantly, more individuals were recorded infected with the disease in the regions. As mentioned in Martins et al. [70] the COVID-19 can be affected by climate and the virus can be spread through respiratory which is the virus moving in the wind movement.

#### The relationship between temperature and humidity

Climatic parameters, such as temperature and humidity, were investigated as the crucial factors in the epidemiology of the respiratory virus survival and transmission of COVID-19 ([61]; S [73, 74].). The rising number of confirmed cases indicated the strong transmission ability of COVID-19 and was related to meteorological parameters. Furthermore, several studies found that the disease transmission was associated with the temperature and humidity of the environment [55, 64, 68, 75], while other investigations have examined and reviewed environmental factors that could influence the epidemiological aspects of Covid-19.

Generally, increased COVID-19 cases and deaths corresponded with temperature, humidity, and viral transmission and mortality. Various studies reported that colder and dryer environments favoured COVID-19 epidemiologically [45, 76, 77]. As example tropical region, the observations indicated that the summer (middle of year) and rainy seasons (end of the year) could effectively diminish the transmission and mortality from COVID-19. High precipitation statistically increases relative air humidity, which is unfavourable for the survival of coronavirus, which prefers dry and cold conditions [32, 34, 78, 79]. Consequently, warmer conditions could reduce COVID-19 transmission. A 1 °C increase in the temperature recorded a decrease in confirmed cases by 8% increase [45].

Several reports established that the minimum, maximum, and average temperature and humidity correlated with COVID-19 occurrence and mortality [55, 80, 81]. The lowest and highest temperatures of 24 and 27.3 °C and a humidity between 76 and 91% were conducive to spreading the virulence agents. The propagation of the disease peaked at the average temperature of 26 °C and humidity of 55% before gradually decreasing with elevated temperature and humidity [78].

Researchers are still divided on the effects of temperature and humidity on coronavirus transmission. Xu et al. [26] confirmed that COVID-19 cases gradually increased with higher temperature and lower humidity, indicating that the virus was actively transmitted in warm and dry conditions. Nevertheless, several reports stated that the spread of COVID-19 was negatively correlated with temperature and humidity [10, 29, 63]. The conflicting findings require further investigation. Moreover, other factors, such as population density, elderly population, cultural aspects, and health interventions, might potentially influence the epidemiology of the disease and necessitate research.

#### Governmental disinfection actions and guidelines

The COVID-19 is a severe health threat that is still spreading worldwide. The epidemiology of the SAR-CoV-2 virus might be affected by several factors, including meteorological conditions (temperature and humidity), population density, and healthcare quality, that permit it to spread rapidly [16, 17]. Nevertheless, in 2020, no effective pharmaceutical interventions or vaccines were available for the diagnosis, treatment, and epidemic prevention against COVID-19 [73, 82]. Consequently, after 2020 the governments globally have designed and executed non-pharmacological public health measures, such as lockdown, travel bans, social distancing, quarantine, public place closure, and public health actions, to curb the spread of COVID-19 infections and several studies have reported on the effects of these plans [13, 83].

The COVID-19 is mainly spread via respiratory droplets from an infected person’s mouth or nose to another in close contact [84]. Accordingly, WHO and most governments worldwide have recommended wearing facemasks in public areas to curb the transmission of COVID-19. The facemasks would prevent individuals from breathing COVID-19-contaminated air [85]. Furthermore, the masks could hinder the transmission of the virus from an infected person as the exhaled air is trapped in droplets collected on the masks, suspending it in the atmosphere for longer. The WHO also recommended adopting a proper hand hygiene routine to prevent transmission and employing protective equipment, such as gloves and body covers, especially for health workers [86].

Besides wearing protective equipment, social distancing was also employed to control the Covid-19 outbreak [74, 87]. Social distancing hinders the human-to-human transmission of the coronavirus in the form of droplets from the mouth and nose, as evidenced by the report from Sun and Zhai [88]. Conversely, Nair & Selvaraj [89] demonstrated that social distancing was less effective in communities and cultures where gatherings are the norm. Nonetheless, the issue could be addressed by educating the public and implementing social distancing policies, such as working from home and any form of plague treatment.

Infected persons, individuals who had contact with confirmed or suspected COVID-19 patients, and persons living in areas with high transmission rates were recommended to undergo quarantine by WHO. The quarantine could be implemented voluntarily or legally enforced by authorities and applicable to individuals, groups, or communities (community containment) [90]. A person under mandatory quarantine must stay in a place for a recommended 14-day period, based on the estimated incubation period of the SARS-CoV-2 [19, 91]. According to Stasi et al. [92], 14-days period for mandatory quarantine it is presenting a clinical improvement after they found 5-day group and 10-day group can be decrease number of patient whose getting effect of COVID-19 from 64 to 54% respectively. This also proven by Ahmadi et al. [43] and Foad et al. [93], quarantining could reduce the transmission of COVID-19.

Lockdown and travel bans, especially in China, the centre of the coronavirus outbreak, reduced the infection rate and the correlation of domestic air traffic with COVID-19 cases [17]. The observations were supported by Sun & Zhai [88] and Sun et al. [94], who noted that travel restrictions diminished the number of COVID-19 reports by 75.70% compared to baseline scenarios without restrictions. Furthermore, example in Malaysia, lockdowns improved the air quality of polluted areas especially in primarily at main cities [95]. As additional, Martins et al. [70] measure the Human Development Index (HDI) with the specific of socio-economic variables as income, education and health. In their study, the income and education levels are the main relevant factors that affect the socio-economic.

A mandatory lockdown is an area under movement control as a preventive measure to stop the coronavirus from spreading to other areas. Numerous governments worldwide enforced the policy to restrict public movements outside their homes during the pandemic. Resultantly, human-to-human transmission of the virus was effectively reduced. The lockdown and movement control order were also suggested for individuals aged 80 and above or with low or compromised immunities, as these groups possess a higher risk of contracting the disease [44].

Governments still enforced movement orders even after the introduction of vaccines by Pfizer, Moderna, and Sinovac, as the vaccines only protect high-risk individuals from the worst effects of COVID-19. Consequently, in most countries, after receiving the first vaccine dose, individuals were allowed to resume life as normal but were still required to follow the standard operating procedures (SOP) outlined by the government.

The government attempted to balance preventing COVID-19 spread and recovering economic activities, for example, local businesses, maritime traders, shipping activities, oil and gas production and economic trades [22, 96]. Nonetheless, the COVID-19 cases demonstrated an increasing trend during the summer due to the higher number of people travelling and on vacation, primarily to alleviate stress from lockdowns. Several new variants were discovered, including the Delta and Omicron strains, which spread in countries such as the USA and the United Kingdom. The high number of COVID-19 cases prompted the WHO to suggest booster doses to ensure full protection.

As mentioned in this manuscript, the COVID-19 still uncertain for any kind factors that can be affected on spreading of this virus. However, regarding many sources of COVID-19 study, the further assessment on this factor need to be continue to be sure, that we ready to facing probably in 10 years projection of solar minimum phase can be held in same situation for another pandemic.

## Discussion

**Fig. 6:**
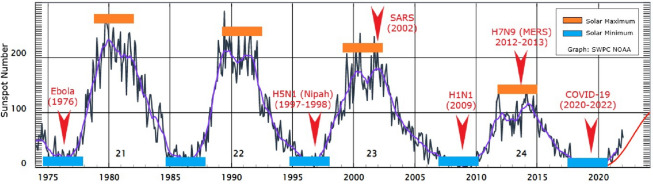
The emergence and recurrence of pandemics every 5 years in relation to solar activities (*Source:* www.swpc.noaa.gov/). *Note:* The yellow boxes indicate the solar maximum, while the blue boxes represent the solar minimum

The sun has an eleven-year cycle known as the solar cycle, related to its magnetic field, which controls the activities on its surface through sunspots. When the magnetic fields are active, numerous sunspots are formed on its surface, hence the sun produces more radiation energy emitted to the earth. The condition is termed solar maximum (see Fig. 6, denoted by the yellow boxes). Alternatively, as the magnetic field of the sun weakens, the number of sunspots decreases, resulting in less radiation energy being emitted to the earth. The phenomenon is known as the solar minimum (see Fig. 6, represented by the blue boxes).

The magnetic field of the sun protects the earth from cosmic or galactic cosmic rays emitted by supernova explosions, stars, and gamma-ray bursts [97]. Nevertheless, galactic cosmic rays could still reach the earth during the solar minimum, the least solar radiation energy period. In the 20th and early 21st centuries, several outbreaks of viral diseases that affected the respiratory system (pneumonia or influenza), namely the Spanish (1918–1919), Asian (1957–1958) and Hong Kong (1968) flu, were documented. Interestingly, the diseases that claimed numerous lives worldwide occurred at the peak of the solar maximum.

Figure 6 illustrates the correlation between the number of sunspots and disease outbreaks from 1975 to 2021, including COVID-19, that began to escalate in December 2019. Under the solar minimum conditions, the spread of Ebola (1976), H5N1 (1997–1998), H1N1 (2009), and COVID-19 (2019-2020) were documented, while the solar maximum phenomenon recorded SARS (2002) and H7N9 (2012–2013) or MERS outbreaks. Nonetheless, solar activity through the production of solar sunspots began to decline since the 22nd solar cycle. Accordingly, further studies are necessary to investigate the influence such solar variations could impart or not on pandemic development.

Despite the findings mentioned above, the sun and cosmic radiations could influence the distribution or outspread of disease-spreading viruses. The rays could kill the viruses via DNA destruction or influence their genetic mutations, which encourage growth and viral evolution. Nevertheless, the connection between radiation and the evolutionary process requires further study by specialists in the field it is become true or not.

The spread of viral diseases transpires naturally in our surroundings and occurs unnoticed by humans. According to records, the spread of pandemic diseases, including the Black Death (fourteenth century) and the Spanish flu (1919), was significantly influenced by the decline and peak of solar activities. Furthermore, in the past 20 years, various diseases related to the influenza virus have been recorded. According to the pattern observed, if all diseases were related to the solar cycle (solar maximum and minimum), the viral diseases would reoccur every 5 to 6 years since they first appeared between 1995 and 2020. Accordingly, the next pandemic might occur around 2024 or 2025 and need to have a proper study for prove these statements. Nonetheless, the activities on the surface of the sun have been weakening since the 23rd solar cycle and it can be proven later after the proper study can be make it.

The beginning of the COVID-19 spread, only several countries with the same winter climate with an average temperature of 5–11 °C and an average humidity rate of 47–79% located at latitudes 30–50 N reported cases. The areas included Wuhan distribution centres in China, the United Kingdom, France, Spain, South Korea, Japan, and the USA (see Fig. 5). Other than biological aspects, the higher number of confirmed cases recorded in colder environments was due to the human body secreting less lymphoproliferative hormone, leading to decreased immunogenicity effects and increased risk of infection [24]. Consequently, the virus could attack and rapidly infect humans during the period [1, 54].

The lymphoproliferative response is a protective immune response that plays a vital role in protecting and eradicating infections and diseases. On the other hand, staying in warm conditions or being exposed to more sunlight would lower the risks of infection. According to Asyary and Veruswati [98], sunlight triggers vitamin D, which increases immunity and increases the recovery rates of infected individuals.

Researchers believe that viruses could survive in the environment for up to 3 to 4 years or even longer. The survival rate of the microorganisms is relatively high, which is related to their biological structures, adaptability on any surfaces, and transmission medium to spread diseases. Viruses possess simple protein structures, namely the spike, membrane, and envelope protein; therefore, when they enter living organisms (such as through the respiratory system), the viruses are easily transmitted.

Once they have entered a host, the viruses duplicate exponentially and swarm the lungs. Subsequently, after the targeted organs, such as the lungs, are invaded, the viruses attack the immune system and create confusion in protective cells to destroy healthy cells. The situation is still considered safe in younger and healthy individuals as their immune systems could differentiate and counter-attack the viruses, curing them. Nonetheless, in elders and individuals with several chronic diseases, most of their protective cells are dead, hence their immune system is forced to work hard to overcome the infection. Pneumonia and death tend to occur when the situation is overwhelming [85]. Consequently, the viruses are harmful to humans as they could multiply in a short period, enter the blood, and overrun the body.

The coronavirus could attach to surfaces without a host, including door knobs and steel and plastic materials. The microorganisms could survive alone, but virologists have yet to determine how long. If someone touches any surface with the virus, the individual would then be infected. The situation would worsen if the infected person contacted numerous people and became a super spreader. A super spreader does not exhibit any symptoms and continuously transmits the virus without realising it. An infected individual transmits the coronavirus via droplets from coughs or sneezes. Nevertheless, scientists have yet to determine if coronavirus is spread via airborne or droplets, hence requiring thorough evaluation [99].

The COVID-19 virus mutates over time, and it can be changing any times. Mutations alter the behaviour and genetic structure of the virus, resulting in a new strain. Numerous research have been conducted to procure vaccines and anti-viral medications, but mutations have led to evolutionary disadvantages. The novel strains are more infectious than the original ones. As of November 2020, approximately six new coronavirus strains have been detected, each displaying different transmission behaviours [100].

Recent studies demonstrated that the mutated viruses exhibit little variability, allowing scientists to produce viable vaccines [71]. Furthermore, different types of vaccines are manufactured by different countries, which could be advantageous. Currently, most countries also recommend booster doses to attain extra protection after receiving the mandatory two vaccine doses. In same time, the social and physical interactions between humans also necessitate to be aware.

The COVID-19 virus is primarily transmitted through droplets produced by an infected person. Accordingly, physical distancing, a one-metre minimum distance between individuals [19], and following the SOP might prevent or avoid spreading the disease. Moreover, self-quarantine, school closures, working from home, cancelling large events, limiting gatherings, and avoiding spending long periods in crowded places are essential strategies in enforcing physical distancing at a community level. The policies are essential precautions that could reduce the further spreading of coronavirus and break the chain of transmission.

Government support also need to control the spread of COVID-19 with the strict SOP. The SOP enforcement in public places would enhance adherence to the new practice among the public and the community, aiding in curbing disease transmission. Practising limited meetings and social gatherings, avoiding crowded places, workplace distancing, preventing non-necessary travels of high-risk family members, especially those with chronic disease, and adhering to the recommended SOP could reduce coronavirus outbreaks. Nonetheless, individual awareness is also necessary to achieve COVID-19 spread prevention.

## Conclusion

Many researchers are focused on identifying the primary drivers of pandemic outbreaks. Seasonal, temperature, and humidity differences significantly impacted COVID-19 growth rate variations. It is crucial to highlight the potential link between the recurrence of pandemics every 5 years and solar activities, which can influence temperature and humidity variations. Notable variations in COVID-19 mortality rates were observed between northern and southern hemisphere countries, with the former having higher rates. One hypothesis suggests that populations in the northern hemisphere may receive insufficient sunlight to maintain optimal vitamin D levels during winter, possibly leading to higher mortality rates.

The first COVID-19 case was detected in Wuhan, China, which is in the northern hemisphere. The number of cases rapidly propagated in December during the winter season. At the time, the temperature in Wuhan was recorded at 13–18 °C. Accordingly, one theory proposes that the survival and transmission of the coronavirus were due to meteorological conditions, namely temperatures between 13 and 18 °C and 50–80% humidity.

Daily rainfall directly impacts humidity levels. The coronavirus exhibited superior survival rates in cold and dry conditions. Furthermore, transmissible gastroenteritis (TGEV) suspensions and possibly other coronaviruses remain viable longer in their airborne states, which are more reliably collected in low relative humidity than in high humidity. Consequently, summer rains would effectively reduce COVID-19 transmission in southern hemisphere regions.

In southern hemisphere regions, the summer seasons are accompanied by a high average temperature at the end and beginning of the year. Countries with temperatures exceeding 24 °C reported fewer infections. As temperatures rise from winter to summer, virus transmission is expected to decline. Nonetheless, the activities and transmission of the virus were expected to decrease during winter to summer transitions, when the countries would be warmer. The peak intensity of infections strongly depends on the level of seasonal transmissions.

Social distancing plays a critical role in preventing the overload of healthcare systems. Many respiratory pathogens, including those causing mild common cold-like syndromes, show seasonal fluctuations, often peaking in winter. This trend can be attributed to increased indoor crowding, school reopening, and climatic changes during autumn.

The spread of COVID-19 to neighbouring regions can be attributed to population interactions. Migration patterns, such as the movement from northern to southern regions during the warmer months, have significant epidemiological impacts. This trend mirrors the behavior of influenza pandemics where minor outbreaks in spring or summer are often followed by major waves in autumn or winter.

## Data Availability

Not applicable.

## Abbreviations

2019-nCoV: Novel coronavirus
COVID-19: Coronavirus disease 2019
DNA: Deoxyribonucleic acid
H1N1: Swine influenza
H5N1: Influenza A virus subtype H5N1
H7N9: Asian Lineage Avian Influenza A(H7N9) Virus
MERS: Middle East respiratory syndrome
MERS-CoV: Middle East respiratory syndrome Coronavirus
PM: Particulate matter
PRISMA: Preferred Reporting Items for Systematic Reviews and Meta-Analyses
ROSES: RepOrting standards for Systematic Evidence Syntheses
SARS: Severe Acute Respiratory Syndrome
SARS-CoV: Severe Acute Respiratory Syndrome Coronavirus
SARS-CoV-2: Syndrome coronavirus 2
SLR: Systematic literature review
SOP: Standard operating procedure
TGEV: Transmissible gastroenteritis Virus
USA: United States of America
WHO: World Health Organization
